# Enamel renal syndrome due to FAM20A mutations: challenging kidney management in view of nephrocalcinosis, hypophosphatemia and hypocalciuria

**DOI:** 10.1186/s13023-026-04232-6

**Published:** 2026-02-05

**Authors:** Marie-Thérèse Eid, Aurélie de Mul, Laure Muresan-Vintila, Laurence Derain Dubourg, Aurélia Bertholet-Thomas, Arnaud Molin, Béatrice Thivichon-Prince, Justine Bacchetta

**Affiliations:** 1 Service de Néphrologie, Rhumatologie et Dermatologie Pédiatriques, Centre de Référence des Maladies Rénales Rares Néphrogones, Centre de Référence des Maladies Rares du Calcium et du Phosphore, Filières Maladies Rares ORKID et OSCAR, Rare Diseases European Networks ERK-Net and BOND, Hospices Civils de Lyon, Lyon, France; 2 Faculté de Médecine Lyon Est, Université Claude Bernard Lyon 1, Lyon, France; 3 Service de Néphrologie, Dialyse, Hypertension et Exploration Fonctionnelle Rénale, Centre de Référence des Maladies Rénales Rares MAREGE, filière maladies rares ORKID, Hôpital E. Herriot, Hospices Civils de Lyon, Lyon, France; 4 Service d’Odontologie, Hospices Civils de Lyon, Lyon, France; 5 INSERM, UMR 1033, Université Claude Bernard Lyon1, Lyon, France; 6 Service de génétique, CHU Caen, Caen, France; 7 Service de Néphrologie, Rhumatologie et Dermatologie Pédiatriques Hôpital Femme Mère Enfant, Boulevard Pinel, Bron Cedex, 69677 France

## Abstract

**Background:**

Enamel Renal Syndrome (ERS) is a rare disorder characterized by a combination of dental and renal abnormalities, including stones and hypophosphatemia. ERS is genetically heterogeneous.

**Methods:**

We report on four pediatric cases of homozygous LoF *FAM20A* mutations (2 families). Biological (including oral calcium load) and imaging (dental and renal) data were reviewed. Results are presented as median(range).

**Results:**

All patients were referred for renal screening by the specialized dental team at a median age of 14.5 [11–19] years. None of them presented symptoms of microscopic/macroscopic hematuria, nor renal colic despite the presence of multiple bilateral nephrolithiasis in all and nephrocalcinosis in one family. Biological parameters were vastly similar, with preserved renal function (eGFR 109(93–111) mL/min/1.73 m²), hypophosphatemia (median − 1.9(-3.4;-1.7) SDS for age), elevated FGF-23 (98(84–117) RU/mL, normal range 21–91 RU/mL) with hypocalciuria and low TmP/GFR. Oral calcium load tests confirmed the absence of resorptive and absorptive hypercalciuria, with adequate PTH inhibition during the test; of note, “baseline” PTH levels tended to be at the upper normal limit (83(65–131) ng/L, local upper normal limit 65ng/L) that was not adequate in view of hypophosphatemia, with 25D levels at 44(19–92) nmol/L. All patients were subsequently followed in pediatric nephrology and received hyperhydration and prudent vitamin D supplementation.

**Conclusion:**

These cases highlight the need for interdisciplinary collaboration between pediatric nephrologists, dental specialists and geneticists, to ensure that patients receive timely renal evaluation. The identification of elevated FGF-23 levels in FAM20A-related ERS with severe nephrolithiasis and hypophosphatemia raises the question of the interest of burosumab as targeted therapy.

**Supplementary Information:**

The online version contains supplementary material available at 10.1186/s13023-026-04232-6.

## Introduction

Enamel renal syndrome (ERS, OMIM#204690) is a rare disorder, genetically heterogeneous (with different genes involved), that intricates renal and oral health. ERS is characterized by different symptoms affecting both the kidneys and the dental enamel, mainly manifesting by a triad of hypoplastic amelogenesis, nephrolithiasis and/or nephrocalcinosis, and hypophosphatemia induced by FGF-23 [1].

Family with sequence similarity 20 member A (*FAM20A*) encodes a Golgi-associated secretory pathway pseudokinase involved in biomineralization and regulation of Fibroblast Growth Factor 23 (FGF-23). It is a trans-membrane protein essential for the phosphorylation of secreted proteins involved in renal and enamel development. In patients with homozygous or compound heterozygous loss of function (LoF) variant in *FAM20A*, oral manifestations are usually evident from early childhood. They include yellow and misshaped teeth, delayed tooth eruption, and intrapulpal calcifications. Nephrocalcinosis is often asymptomatic but can progress during late childhood or early adulthood to impaired renal function, recurrent urinary infections, and renal tubular acidosis. Chronic kidney disease (CKD) was even reported, with dialysis requirement in exceptional cases [2, 3]. Kidney biopsies of patients with *FAM20A* homozygous LoF variant showed the presence of focal clusters of sclerosed glomeruli, marked periglomerular fibrosis with lymphocytic and plasma cell infiltration of the renal interstitium. Hypocalciuria, hypocitraturia and renal phosphate loss are usually described in patients with *FAM20A* homozygous LoF variant. Supplemental Table 1 illustrates the current knowledge on renal phenotype in patients with ERS. In those where genetic testing was realized, *FAM20A* LoF variants were detected [4, 5].

In our university hospital, a systematic approach has been chosen since 2022: indeed, we perform a systematic non-invasive renal screening by renal ultrasounds in all patients with amelogenesis imperfecta (AI) who are referred by the expert center for rare dental diseases to the pediatric nephrology department. A total of 30 AI patients has been evaluated, and we have found six patients with kidney involvement, i.e., four patients with *FAM20A* variants from 2 different consanguineous families and two patients with *WDR72* variants. Here, we highlight specificities in the *FAM20A* tubular phenotype that raises questions on a potential future therapy targeting FGF23 in a drug-repurposing approach.

## Cases

This retrospective series of cases was approved by the local ethical committee (*Comité d’Ethique des Hospices Civils de Lyon*, AGORA 25-5073, session April 30th, 2025), and the work is in accordance with the declaration of Helsinki. All patients gave oral consent for publication of dental pictures. Clinical parameters (such as age, sex, age at first symptoms, age at referral to pediatric nephrology, renal symptoms, growth), biological data (including estimated glomerular filtration rate, eGFR, with the 2009 Schwartz formula, mineral parameters and notably age-normalized phosphate, PTH, 25-OH vitamin D, 1,25-(OH)_2_ vitamin D, FGF23, total alkaline phosphatase, urinary calcium, urinary citrate, tubular phosphate handling with the calculation of TmP/GFR and oral calcium load, as previously described as well as imaging results (both dental and renal) were reviewed (Table 1). Of note, three patients had bone examination by DXA, and no overt bone phenotype was found.

**Table 1:** Summary of patients with FAM20A homozygous mutation

	Patient 1		Patient 2		Patient 3		Patient 4
Family	1		1		1		2
Sex	Male		Female		Female		Female
Age of first dental symptom	Tooth eruption		Tooth eruption		Tooth eruption		4 years
Variant in *FAM20A* (NM_017565.4)	c.1023_1024insTT p.(Ala342LeufsTer4l) in exon 7		c.1023_1024insTT p.(Ala342LeufsTer4l) in exon 7		c.1023_1024insTT p.(Ala342LeufsTer4l) in exon 7		c.813–2 A > G; p.? in intron 5
Associated dental anomaly	Severe hypoplastic amelogenesis imperfecta, pulp calcifications, root dilaceration, dental follicle hyperplasia of impacted teeth, gingival hyperplasia		Severe hypoplastic amelogenesis imperfecta, pulp calcifications, gingival hyperplasia		Severe hypoplastic amelogenesis imperfecta, pulp calcifications, root dilaceration, dental follicle hyperplasia of impacted teeth, gingival hyperplasia		Severe hypoplastic amelogenesis imperfecta, pulp calcifications, root dilaceration, dental follicle hyperplasia of impacted teeth, gingival hyperplasia
Dental phenotype							Not available
Past renal medical history	No		No		No		No
Microscopic or macroscopic hematuria	No		No		No		No
Presence of renal colic	No		No		No		No
Nephrocalcinosis (grade)	Yes (grade II)		Yes (grade III)		Yes (grade II)		No
Nephrolithiasis	Calyceal left kidney stone of 6 mm, calyceal right kidney stone of 4 mm		Calyceal left and right kidney stones + parapyelic cysts		Calyceal left and right kidney stones		Calyceal left and right kidney stones
Renal Ultrasound							
Age at nephrology referral (years)	16		19		13		11
Height (cm)	173		159		170		151
Normal growth	Yes		Yes		Yes		Yes
Creatinine (µmol/l)	57		48		45		53
eGFR (ml/min/1.73 m²)	111		108		123		93
Sodium (mmol/L)	141		139		138		140
Potassium (mmol/L)	4.0		3.7		3.9		4.0
Bicarbonate (mmol/L)	25		24		22		22
Calcium (mmol/L)	2.33		2.36		2.23		2.37
Phosphate (mmol/L)	1.16 ↓		0.72 ↓		1.14 ↓		1.23 ↓
SDS phosphate for age	-1.7		-3.4		-2.0		-1.8
Hypophosmatemia for age (Yes/No)	Yes		Yes		Yes		Yes
Magnesium (mmol/L)	0.82		0.74		0.89		0.79
Total ALP (UI/L)	292		115		217		447
PTH (ng/L) (N 15–65)	65 ↑		85 ↑		81 ↑		131 ↑
C terminal FGF-23 (Ru/ml)	117 ↑		93 ↑		103 ↑		84 ↑
25-OH vitamin D (nmol/L)	45 ↓		43 ↓		19 ↓		92
1,25-(OH)_2_ vitamin D (pmol/L) (*N* < 200)	165		102		100		191
Urinary calcium(mmol/kg/24hr)	0.03 ↓		0.02 ↓		0.01 ↓		NA
Urinary Calcium/Creatinine (mmol/mmol)	0.04 ↓		0.15 ↓		0.08 ↓		0.06 ↓
Urinary Oxalate/Creatinine (mmol/mmol) (*N* < 0.08)	0.05		0.03		0.05		0.07
TmP/GFR(mmol/L)	1.06		0.78		1.34		1.16
Renal phosphate leak (Yes/No)	Yes		Yes		Yes		Yes
Urinary Citrate (mmol/L)	1.38		1.27		0.76		1.03
Urinary Citrate/Creatinine (mmol/mmol)	0.24		0.24		0.33		0.29
Urinary Calcium/Citrate (mmol/mmol)	1.2		0.9		0.26		0.2
Urinary pH	5.0		6.4		5.6		6.6
Cristalluria	Negative		Negative		Negative		Negative
Baseline of calcium load: Urinary Calcium/Creatinine mmol/L / PTH ng/L	0.04	65	0.14	70	0.08	72	
2 h after calcium load: Urinary Calcium/Creatinine mmol/L / PTH ng/L	0.06	32	0.28	36	0.27	29	
4 h after calcium load: Urinary Calcium/Creatinine mmol/L / PTH ng/L	0.11	52	0.49	42	0.47	48	
Conclusion of calcium load test	Absence of resorptive and absorptive hypercalciuria	PTH well inhibited by the calcium load test	Absence of resorptive and absorptive hypercalciuria	PTH well inhibited by the calcium load test	Absence of resorptive and absorptive hypercalciuria	PTH well inhibited by the calcium load test	

eGFR: estimated glomerular filtration rate

SD : standard deviation score

ALP: alkaline phosphatase

PTH: parathyroid hormone

N: local normal values

TmP/GFR: tubular maximum phosphate reabsorption per glomerular filtration

All patients with *FAM20A* homozygous variants were referred for renal screening by the specialized dental team at teenage years. None of them presented symptoms of microscopic/macroscopic hematuria, nor renal colic despite the presence of multiple bilateral nephrolithiasis in all, associated with nephrocalcinosis in Family 1 (3 patients). Biological parameters were vastly similar in all patients: they mainly presented with preserved renal function, hypophosphatemia, low TmP/GFR and moderately elevated FGF23. Despite significant nephrolithiasis in size and number, hypocalciuria was constant, further confirmed by oral calcium load tests with the absence of resorptive and absorptive hypercalciuria and adequate PTH inhibition during the test. Interestingly, baseline PTH levels tended to be at the upper normal limit, despite adequate nutritional calcium intake, normal 25-OH vitamin D levels and normal calcium levels. Citraturia was in the lower normal range. Symptomatic management was proposed, including oral hyperhydration, nutritional advice (diet controlled in sodium and proteins with normal calcium intake for age) and correction of 25-OH vitamin D deficiency (if necessary) during follow-up using low daily doses of nutritional vitamin D. We also propose a description of patients with WDR72 LoF variants in Supplemental Table 2.

## Discussion

Our findings underscore the need for interdisciplinary collaborations, particularly between dentists, pediatric nephrologists and geneticists in this ultra-rare disease (prevalence estimated < 1/1,000,000).

Since 2022, the Lyon dental expert center on rare dental diseases systematically refers patients with amelogenesis imperfecta for a non-invasive renal screening consisting of renal ultrasounds performed by expert pediatric radiologists [6]. In case of abnormal renal US, a biological evaluation is performed and a clinical follow-up is organized. One question that remains open is whether the absence of abnormalities on the first renal US can completely rule out any renal involvement in the future: since epidemiological data are scarce, we propose performing a renal US every 5 years in ERS patients seen in pediatric nephrology with normal renal US. Kidney tomography may be more sensitive, but in clinical routine, we avoid unnecessary radiation exposure, as recommended by pediatric radiologists [6]. Moreover, we advise parents to follow general “bone-protective” measures that should be applied to all children between 3 and 18 years (that seem crucial in children with enamel problems), namely normal calcium intake for age (i.e., 3 to 4 dairy products per day) and native vitamin D supplementation, as recommended in the 2022 French guidelines in general pediatrics.

**Fig. 1:**
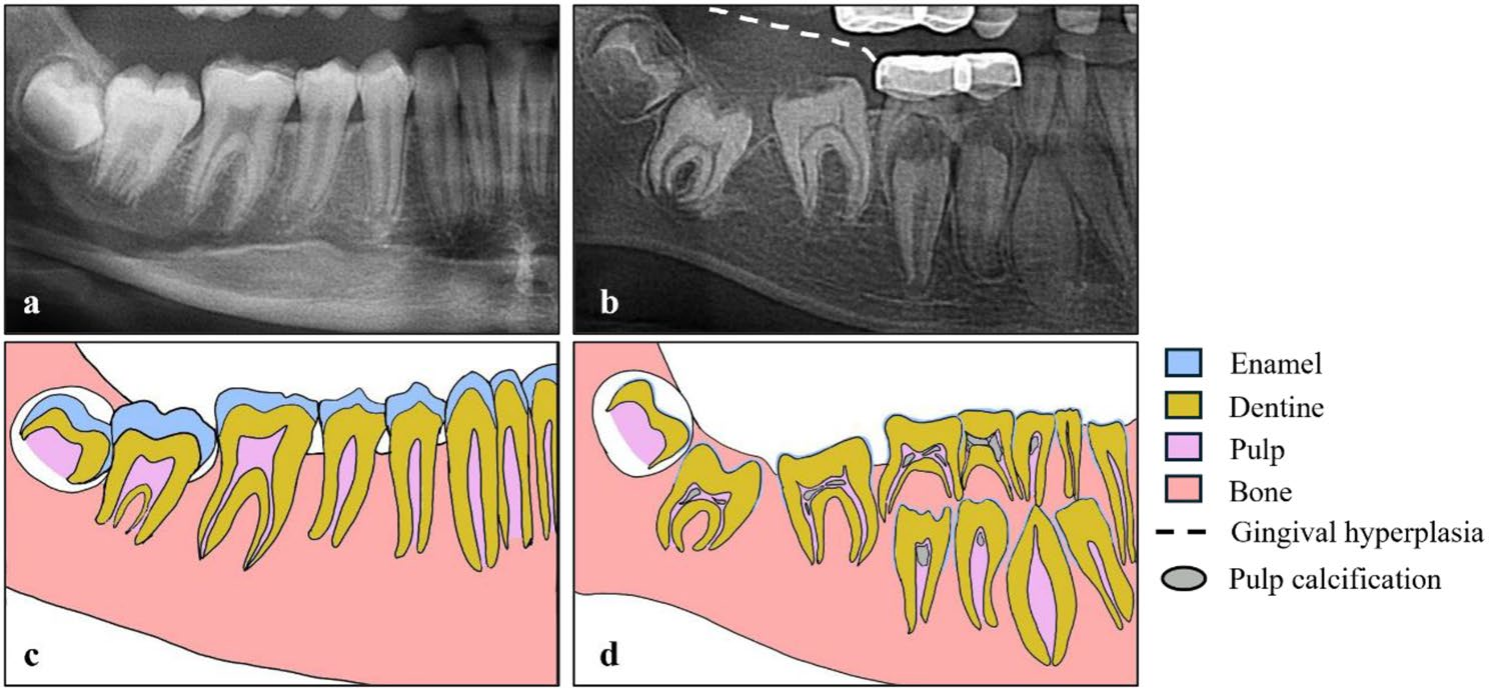
Radiological signs on the orthopantomogram that can be compatible with FAM20A mutations. Radiographic and schematic views show radiological signs in an 11-year old ERS patient (**b**,**d**) compared to an age-matched healthy control (**a**,**c**). Pathognomonic radiological oral signs in ERS patient include hypoplastic/absence of enamel, flat cusps of posterior teeth, delayed tooth eruption, pulp calcification (**b**,**d**), and gingival hyperplasia (**b**). All signs are generalized throughout the oral cavity. The preformed pediatric crowns visible on the radiograph (**b**) are not shown in the diagram (**d**)

All patients diagnosed in Lyon with *FAM20A* homozygous variants displayed nephrolithiasis and sometimes nephrocalcinosis, whereas they were not symptomatic. Without systematic renal screening, they would have been diagnosed at the time of a renal complication, when nephrocalcinosis and lithiasis have progressed and maybe already induced CKD. However, an early diagnosis of renal complications is crucial to tailor management, using nutrition, hyperhydration and symptomatic therapies (e.g., correction of native vitamin D deficiency) as first line. Of note, the presence of renal phosphate leak associated with profound hypocalciuria prevented us to give citrate, and the absence of hypercalciuria prevented us to prescribe thiazides. Specific dental abnormalities (such as hypoplastic amelogenesis imperfecta associated with multiple calcification nodules in pulp chambers and delayed/impaired dental eruption, Fig. 1) should prompt the dentist to perform a genetic testing and to refer the patient to a pediatric nephrologist.

In terms of renal tubular phenotype associated with *FAM20A* variants, we confirm the previously described findings: hypocalciuria, hypocitraturia, renal phosphate loss and slightly increased FGF23 levels. Moreover, we performed a complete and exhaustive lithiasis work-up in our pediatric renal physiology unit (to rule out other causes of lithiasis) as well as an oral calcium load confirming baseline hypocalciuria and the absence of hypercalciuria (whether absorptive or resorptive) during the dynamic test. Because patients did not pass stones, we could unfortunately not provide stone analysis. In animal models, *Fam20a*^*–/–*^ mice develop severe AI and disseminated calcifications of muscular arteries and intrapulmonary calcifications; however, they have normal calcium and phosphate levels with normal dentin and bone. These mice were also found to have widespread and severe ectopic calcification in the kidneys: 2/3 of them had small kidneys with widespread calcification affecting virtually all small to medium size muscular arteries on histological examination [7].

Fam20a is expressed in ameloblasts, odontoblasts, pulmonary and parathyroid cells, with local and systemic effects suggesting both local and/or systemic effects for FAM20A, but there is no mention of FAM20A expression in tubular renal cells in the literature [7].

The mechanisms behind increased FGF23 levels in patients or animal models with FAM20A deficiency remains to be fully defined. The answer could be in the interaction between FAM20A and FAM20C. Indeed, FAM20C, whose deficiency is responsible for autosomal recessive Raine syndrome (OMIM#259775) and hypophosphatemic rickets both in humans and in mice [8], is a direct inhibitor of FGF23 [9]. FAM20A interacts with FAM20C to control FAM20C localization and allow its extracellular function in mineralized tissues on the other hand [10]. Thus, LoF variants in *FAM20A* likely impairs FAM20C action, and indirectly increases FGF23 levels, with subsequent hypophosphatemia [11]. That said, mechanisms behind nephrolithiasis/nephrocalcinosis in these patients remain poorly understood since it is highly unlikely that hypophosphatemia alone induces such renal ectopic mineralization. Hypercalciuria is not the mechanism by which patients develop nephrolithiasis; we even show deep hypocalciuria both at baseline and after dynamic tests, hence thiazides cannot be proposed. In contrast, hypocitraturia may partly explain the risk of nephrolithiasis, but its mechanisms are unknown and the correction of hypocitraturia by citrate supplementation may increase urinary pH and therefore increase the risk of phosphate stones in this setting of renal phosphate leak.

Still, these four patients were teenagers with quite severe renal phenotype on the US. With the risk of evolution towards CKD and even kidney failure [2, 3], and with a tubular phenotype of secondary renal phosphate leak (i.e., hypophosphatemia, low TmP/GFR, and slightly increased FGF23 levels), it is tempting to propose antiFGF23 therapies in these patients, for example the fully human monoclonal anti-FGF23 antibody burosumab, even in the absence of obvious rickets (as indicated by normal alkaline phosphatase levels). Indeed, the only mechanism that can explain hypophosphatemia here is increased FGF-23 levels, because we can rule out all other causes of hypophosphatemia. First, patients with hypophosphatemia due to nutritional deficiency or intestinal malabsorption display normal or even increased Tmp/GFR. Second, patients here do not present primary hyperparathyroidism in the absence of hypercalcemia and high PTH levels. Last, primary renal phosphate leak rather induces low FGF-23 levels. Thus, theoretically, proposing an anti FGF23 therapy may normalize urine phosphate leak, and allow us to add citrate for the renal part of the disease. This drug is approved in children older than 1 year in Europe, suffering from X-linked hypophosphatemia, and is well tolerated [12, 13]. Further research is necessary to establish its interest in this indication, but could be a solution in the most severe patients. However, to date, national regulations do not allow physicians to treat such patients.

## Conclusion

These cases of ERS highlight the need for interdisciplinary collaborations between pediatric nephrologists, dental specialists and geneticists. Raising awareness on the systemic implications of dental abnormalities is crucial to early identify patients with renal impairment and to ensure personalized management. The presence of multiple, recurrent and big stones in size with nephrocalcinosis in view of significant hypophosphatemia with slightly increased FGF23 levels raises the question of proposing targeted therapies in these young patients with *FAM20A* homozygous mutations.

## Supplementary Information

Below is the link to the electronic supplementary material.

Supplementary Material 1

## Data Availability

The data that support the findings of this study are available on request from the corresponding author.

## Abbreviations

ERS: Enamel renal syndrome
FAM20A: Family with sequence similarity 20 member A
WDR72: WD repeat-containing protein 72
FGF23: Fibroblast growth factor 23
CKD: Chronic Kidney Disease
LoF: Loss of Function
