# Supplementary material for: Enamel renal syndrome due to FAM20A mutations: challenging kidney management in view of nephrocalcinosis, hypophosphatemia and hypocalciuria
Source: Orphanet J Rare Dis. 2026 Feb 5;21:90. doi: 10.1186/s13023-026-04232-6 (PMC12969917; doi:10.1186/s13023-026-04232-6)
Supplement: Supplementary file 1 — Supplementary Material 1 [file 13023_2026_4232_MOESM1_ESM.docx]

**Supplemental Table 1: Literature Review on Enamel Renal Syndrome (ERS) with different renal phenotypes**

| **Authors** | **Year** | **Number of patients** | **Renal phenotype** | **Genetic description** | **Reference number** |
| --- | --- | --- | --- | --- | --- |
| MacGibbon et al | 1972 | 2 | Hypertension  Acute and chronic pyelonephritis  Nephrocalcinosis that progressed to renal failure  Normal calcium phosphorus metabolism | Not described | (14) |
| Lubinsky et al | 1985 | 2 | Enuresis  Intermittent urinary infections  Nephrocalcinosis  Normal calcium phosphorus metabolism | Not described | (15) |
| Hall et al | 1995 | 2 | Nephrocalcinosis  Normal calcium and phosphorus metabolism  Renal biopsy showed focal clusters of sclerosed glomeruli, although most glomeruli appeared normal. The predominant feature was infiltration of the interstitium with lymphocytes and plasma cells with marked periglomerular fibrosis. | Not described | (16) |
| Dellow et al | 1999 | 2 | Polyuria and polydipsia  Nephrocalcinosis  Chronic pyelonephritis  Multiple renal cysts  Renal failure | Not described | (17) |
| Paula et al | 2005 | 1 | Nephrocalcinosis | Not described | (18) |
| Jaureguiberry et al. | 2013 | 25 | Nephrocalcinosis | FAM20A mutation:  c.915-918delCTTT; p.F30515X380  IVS2+1G>A/c.913-914delTT; p.F30515X378  IVS4+1G>C/c.1348-1349delTC; p.S45018X469  c.1475-1482dupAACCCCAG; p.L49518X509  c.406C>T; p.R136X  c.34-35delCT; p.L12fsX78  c.1513delA; p.I505fsX506  c.1432C>T; p.R478X  c.518T>G; p.L173R  c.727C>T/c.1228-1229delGA; p.R243X/p.D410fsX414  c.217C>T/c.727C>T; p.R73X/p.R243X  c.1369A>T; p.K457X  c.755-757delTCT/c.641-719del79bp; p.F252del/p.I214fsX259  IVS5+2T>G  c.907-908delAG; p.S303fsX378  c.34-35delCT/c.612delC; p.L12fsX78/p.A204fsX215 | (19) |
| Kantaputra et al. | 2013 | 2 | Renal nephrocalcinosis and hyperechogenecity  Nephrolithiasis | - homozygous novel c.34_35delCT mutation in exon 1 of FAM20A - homozygous novel c.1482_1483insAC mutation in exon 11of FAM20A | (20) |
| Wang et al. | 2013 | 3 | Nephrocalcinosis detected in one patient | - FAM20A mutation c.992G>A; g.63853G>A; p.G331D. - FAM20A mutation c.720-2A>G; g.62232A>G; p.Q241_R271del. - FAM20A nonsense mutations in exon 2 (c.406C>T; g.50213C>T; p.R136*) and in exon 11 (c.1432C>T; g.68284C>T; p.R478*) | (21) |
| Ashkenazi et al | 2014 | 1 | Macroscopic hematuria  Nephrocalcinosis  Hypocitraturia  Renal calcifications and obsructions in the renal transmission canaliculi  Deterioration to renal failure | Not described | (3) |
| Pêgo et al | 2017 | 2 | Nephrocalcinosis  Normal calcium and phosphorus metabolism  Kidney failure | Homozygous FAM20A for a c.406 C>T nonsense mutation in exon 2 that caused a premature termination at p.Arg136 | (2) |
| Debnath et al | 2019 |  | Agenesis of the kidney | Not described | (22) |

**Supplemental Table 2:** Description of the two patients (same family) with WDR72 LoF mutations

|  | **Patient 1** | | **Patient 2** | |
| --- | --- | --- | --- | --- |
| Sex | Female | | Male | |
| Age of referral to nephrology | 4 years | | 1 year | |
| Variant in WDR72 (NM 182758.4) | 2 variations at the  heterozygous state in exons 3 and 9  c.[237C>A(;)932del],  p.[(Tyr79*)  (;) (Ser311Leufs*19)]. | | 2 variations at the heterozygous state in exons 3 and 9  c.[237C>A(;)932del], p.[(Tyr79*) (;) (Ser311Leufs*19)]. | |
| Age of first dental symptom | At tooth eruption | | At tooth eruption | |
| Presence of microscopic or macroscopic hematuria (Yes/no) | No | | No | |
| Presence of renal colic (Yes/no) | No | | No | |
| Consanguinity | No | | No | |
|  |  | |  | |
| Past renal medical history | None | | None | |
| Nephrocalcinosis  Grading | Yes  Grade II | | No | |
| Nephrolithiasis | No | | No | |
| Renal Ultrasound | U/S not available at our center | | 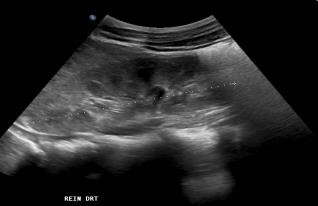 | |
|  |  | |  | |
| Height (cm) | 127 | | 168 | |
| Normal Growth | Yes | | Yes | |
| Creatinine (µmol/l) | 43 | | 46 | |
| eGFR (ml/min/1.73m²) | 108 | | 133 | |
| Sodium (mmol/L) | 140 | | 139 | |
| Potassium (mmol/L) | 4 | | 3.8 | |
| Bicarbonate (mmol/L) | 17 **↓** | | 22 **↑** | |
| Calcium (mmol/L) | 2.58 | | 2,5 | |
| Phosphorus (mmol/L) | 1.64 | | 1,61 | |
| Hypophosmatemia for age (Yes/No) | No | | No | |
| SD phosphorus | -0.15 | | 0.6 | |
| Magnesium (mmol/L) | 0.99 | | 0,94 | |
| ALP (UI/L) <500 | 330 | | 327 | |
| PTH (ng/L) (15-65) | 26 | | 48 | |
| FGF-23 (Ru/ml) (21-91) | 81.4 | | 293 ↑ | |
| 25-OH vitamin D (nmol/L) | 64 **↓** | | 45 **↓** | |
| 1,25-OH vitamin D (nmol/L) | 194 | | 281 | |
| Calciuria mmol/kg/24hr | 0.07 **↓** | | 0.01**↓** | |
| Urinary Calcium/Creatinine (mmol/mmol) | 0.41 | | 0.09 **↓** | |
| Urinary Oxalate/Creatinine | Normal | | Not available | |
| TmP/GFR(mmol/L) | 1.51 | | 1.57 | |
| Renal phosphate leak (Yes/No) | No | | No | |
| Citrate (mmol/L) | 0.28 **↓** | | Too low to be measured | |
| Citrate/Creatinine (Urinary) | 0.05 **↓** | | Too low to be calculated | |
| Urinary pH | 6.4 | | 7 | |
| Crystalluria | Negative | | Negative | |
| Calcium/Creatinine (urinary) before calcium load/PTH | 0.41 | 26 | 0.02 | 48 |
| Calcium/Creatinine (urinary) 2 hours after calcium load /PTH | 0.88 | 21 | 0.06 | 58 |
| Calcium/Creatinine (urinary) 4 hours after calcium load/ PTH | 0.48 | 27 | 0.08 | 52 |
| Conclusion of calcium load test | Absence of resorptive and absorptive  hypercalciuria    Calciuria before calcium load test slightly elevated | Inhibition of PTH not analysed due to the unkown decrease in calcium after the calcium load test | Absence of resorptive and absorptive hypercalciuria | Inhibition of PTH not analysed due to the unkown decrease in calcium after the calcium load test |
